# Longitudinal analysis of sinus microbiota post endoscopic surgery in patients with cystic fibrosis and chronic rhinosinusitis: a pilot study

**DOI:** 10.1186/s12931-021-01697-w

**Published:** 2021-04-13

**Authors:** Brett Wagner Mackenzie, Camila Dassi, Anitha Vivekanandan, Melissa Zoing, Richard G. Douglas, Kristi Biswas

**Affiliations:** Department of Surgery, The University of Auckland, 85 Park Road, Grafton, 1023 Auckland, New Zealand

**Keywords:** Cystic fibrosis, Chronic rhinosinusitis, Antimicrobial resistance, Sinonasal microbiota, Cultivation

## Abstract

**Background:**

Cystic fibrosis is a debilitating, autosomal recessive disease which results in chronic upper and lower airway infection and inflammation. In this study, four adult patients presenting with cystic fibrosis and chronic rhinosinusitis were recruited. Culture and molecular techniques were employed to evaluate changes in microbial profiles, host gene expression and antimicrobial resistance (AMR) in the upper respiratory tract over time.

**Methods:**

Swab samples from the sinonasal cavity were collected at the time of surgery and at follow-up clinics at regular time intervals for up to 18 months. Nucleic acids were extracted, and DNA amplicon sequencing was applied to describe bacterial and fungal composition. In parallel, RNA was used to evaluate the expression of 17 AMR genes and two inflammatory markers (interleukins 6 and 8) using custom qPCR array cards. Molecular results were compared with routine sinus and sputum culture reports within each patient.

**Results:**

Bacterial amplicon sequencing and swab culture reports from the sinonasal cavity were mostly congruent and relatively stable for each patient across time. The predominant species detected in patients P02 and P04 were *Pseudomonas aeruginosa*, *Staphylococcus aureus* in patient P03, and a mixture of *Enterobacter* and *S. aureus* in patient P01. Fungal profiles were variable and less subject specific than bacterial communities. Increased expressions of interleukins 6 and 8 were observed in all patients throughout the sampling period compared with other measured genes. The most prevalent AMR gene detected was *ampC*. However, the prevalence of AMR gene expression was low in all patient samples across varying time-points.

**Conclusions:**

We observed a surprising degree of stability of sinonasal microbial composition, and inflammatory and AMR gene expression across all patients post sinus surgery.

**Supplementary Information:**

The online version contains supplementary material available at 10.1186/s12931-021-01697-w.

## Background

Cystic fibrosis (CF) is the most common life-threatening inherited condition in Caucasian populations and results in the build-up of thick mucus, and repeated cycles of airway infection. [1]. Although bacterial lung infections ultimately shorten the life expectancy in most CF patients, nearly all CF patients also suffer from long-term inflammation of the sinuses, or chronic rhinosinusitis (CRS) [2]. The unified airway theory proposes a bi-directional relationship between the upper and lower airway microbiota [3]. Recent evidence suggests that the sinuses can serve as a reservoir for bacterial pathogens that subsequently colonise the lungs [4, 5]. Furthermore, medical and surgical management of upper respiratory inflammation is associated with improved lower respiratory outcomes [6, 7].

The upper and lower airways of adult CF patients are typically dominated by potentially pathogenic bacteria such as *Staphylococcus aureus*, *Pseudomonas aeruginosa*, *Haemophilus* spp., *Streptococcus* spp., *Achromobacter* spp., *Burkholderia* spp., and *Enterobacter* spp*.* [8–12]. Similar bacteria can be detected in the lungs of healthy adults, however, bacterial diversity is significantly increased, meaning more bacterial species are detected, and the dominance of a single species is not observed [13]. When comparing adult CF patients experiencing pulmonary acute exacerbations to those that are clinically stable, the bacterial load of the dominant bacterial pathogen increases [14]. In addition to an increased colonisation of pathogens, the airways of CF patients also have significantly increased expression of inflammatory markers and extensive airway remodelling compared to healthy controls [15, 16].

The polymicrobial infections associated with the lower airways of CF patients have been extensively researched [17–20]. However, several aspects of the CF-associated upper airway microbiota, including its temporal stability, relationship to the lower airway microbiota, and contribution to acute exacerbation events, remains less well defined. Recent research suggests that the sinus microbiota in CF-CRS patients are distinct from those patients with idiopathic CRS and are characterized by a significant decrease in overall bacterial diversity and richness accompanied by an increase in bacterial load [10]. Concordant results have also been observed in the lower airways of CF patients when compared with aged-matched, healthy patients [9]. When comparing healthy and idiopathic CRS sinonasal microbial community compositions to patients with CF, significant increases in relative abundance and prevalence of *Corynebacterium* spp., *Anaerococcus* spp., and *Propionibacterium* spp. are observed in the former [10].

Mitigating lung function decline is one of the main goals of CF treatment. Even with the significant improvements in modern treatment, such as ivacaftor, most CF patients will require multiple courses of oral and intravenous antibiotics. As the disease progresses patients often require more intensive antibiotic therapy. This lifelong exposure to antibiotics is associated with the development of antimicrobial resistance (AMR) and a loss of bacterial diversity. An exemplar longitudinal study focusing on the lower airway microbiota in CF patients found that antibiotic use was the most significant driver of reduced bacterial diversity [21].

One recent study suggested that CRS exacerbations may be a precursor to pulmonary exacerbations [22]. CRS exacerbations can be caused by viral or bacterial infections and are characterised by an acute and transient worsening of symptoms [23]. Understanding the composition of the CF-CRS sinonasal microbiota, congruence between cultivation and molecular results, and the accumulation and expression of AMR genes are critical for improving treatment to reduce lung function decline. In our pilot study, we describe the stability of the sinonasal microbiota in four adult CF-CRS patients up to 18 months post sinus surgery. We also measured the expression of inflammatory marker genes and a range of AMR genes. These molecular data were reported in conjunction with patient clinical and culture data for both sputum and sinus swab samples.

## Methods

The objective of this pilot study was to describe the sinonasal microbiota, and host inflammatory and AMR gene expression in adult cystic fibrosis patients post endoscopic sinus surgery during clinical stability. A combination of cultivation and molecular techniques were applied in this investigation.

### Patient data and sample collection

Four subjects undergoing functional endoscopic sinus surgery (FESS) for extensive bilateral CRS were recruited for this study. Patients aged > 18 years and with a diagnosis of both CRS and CF were included in this study. Patients were diagnosed with CF prior to this study and were subsequently diagnosed with CRS according to the 2012 European Position Paper definition [24]. Three patients were homozygous for Δf508 mutation and one patient was diagnosed with CF based on the evidence of phenotypic disease and sweat chloride test results > 60 mmol/L. Extensive demographic and clinical data were collected from the patients in this study (Table 1), including long-term medications and recent antibiotic prescription data (Additional file 1: Tables S1, S2).

**Table 1:** Demographic and clinical data collected from the four patients in this study

Patient	Collection timepoint^a^	Sample collection procedure	Age^b^	Sex	Smoker	Ethnicity	CF diagnostic criteria	Age at CF diagnosis	Age at CRS diagnosis	Lung transplant	Nasal polyposis	Lund-Mackay
P01	Baseline	FHFESS	21	M	No	NZE	Δf508	< 5	11	No	Yes	21
	6	Clinic										
	13	Clinic										
	15	Clinic										
	18	FHFESS—revision										
P02	Baseline	FHFESS	36	F	No	NZE	Δf508	< 5	Unknown	No	Yes	12
	2	Clinic										
	6	Clinic										
	11	Clinic										
P03	Baseline	FHFESS	50	F	Ex	Maori	Positive sweat tests, no CFTR mutation on genotyping^c^	> 18	42	No	Yes	13
	3	Clinic										
	6	Clinic										
	12	Clinic										
P04	Baseline	FHFESS—revision	31	F	No	NZE	Δf508	< 5	21	2012	Yes	19
	3	Clinic										
	6	Clinic										
	7	FHFESS—revision										
	11	Clinic										

*CF* cystic fibrosis, *CRS* chronic rhinosinusitis, *FHFESS* full house functional endoscopic sinus surgery, *NZE* New Zealand European

^a^Collection timepoint refers to months from baseline sample collection

^b^Age at baseline sample collection

^c^Fulfils diagnostic criteria on the grounds of appropriate phenotypic features (chronic sinopulmonary disease) and laboratory evidence of CFTR dysfunction (sweat chloride > 60 mmol/L on two separate occasions)

As part of standard clinical care, a nasal swab and sputum sample were collected for routine cultivation analyses at the hospital laboratory. In addition to samples for culture, sterile rayon-tipped swabs (Copan, #170KS01) were collected under endoscopic guidance from the left and right middle meatuses by a single surgeon for molecular analyses. After collection, swabs were stored in RNAlater^®^ preservative overnight at 4 °C, then transferred to − 80 °C until processing. Baseline samples were collected during FESS, and follow-up samples were collected in clinic at regular time intervals up to 18 months postoperatively. If a patient required revision FESS, additional samples were collected.

### Sample processing for cultivation

Nasal swabs and sputum samples were processed for routine microbiological culture at Auckland City Hospital LabPLUS laboratory. Relevant pathogens and significant isolates were identified by colony morphology, with further species classification using matrix-assisted laser desorption ionization time-of-flight mass spectrometry (VITEK^®^ MS, Biomerieux). Other groups of organisms that were detected but not identified as clinically relevant included skin flora from nasal swabs, mixed oropharyngeal flora in sputum or nasal swabs, mixed Gram-positive and Gram-negative bacteria. Quantitative assessments of bacterial colonies were not collected. Positively identified colonies were assessed for antibiotic resistance and susceptibility profiles according to EUCAST criteria. Results were interpreted from reports generated by the laboratory.

### Sample processing for bacterial and fungal amplicon sequencing

DNA and RNA were extracted from pairs of sterile rayon-tipped swabs using the Qiagen AllPrep DNA/RNA Mini Kit as previously described [10]. The baseline samples from all four patients and the 7-month post-surgery sample from patient P04 were processed in previous studies [10, 25]. The DNA extracted from these samples were used to extinction and excluded from fungal ITS2 amplicon sequencing.

Extracted DNA was used for PCR amplification and purification of the V3–V4 regions of the bacterial 16S rRNA gene and fungal ITS2 as previously described [26]. The V3–V4 primers, including Illumina adaptors in underlined typeface, are as follows: S-D-Bact-0341-b-S-17 5′-TCGTCGGCAGCGTCAGATGTGTATAAGAGACAG-CCTACGGGNGGCWGCAG-3′ and S-D-Bact-0785-a-A-21 5′-GTCTCGTGGGCTCGGAGATGTGTATAAGAGACAG-GACTACHVGGGTATCTAATCC-3′. The ITS2 primers were as follows: ITS3 5′-TCGTCGGCAGCGTCAGATGTGTATAAGAGACAG-GCATCGATGAAGAACGCAGC-3′ and ITS4 5′-GTCTCGTGGGCTCGGAGATGTGTATAAGAGACAG-TCCTCCGCTTATTGATATGC-3′. Purified amplicons were sequenced by Auckland Genomics on the Illumina MiSeq using 2 × 300 bp paired-end sequencing.

### Bioinformatic processing of amplicon data

Bacterial and fungal amplicon sequencing data were processed using usearch v10.0.240 [27, 28]. Zero-radius operational taxonomic units (zOTUs) of unique biological replicates were generated for both bacterial and fungal data to improve differentiation of closely related sequences. The bacterial (https://github.com/mhog025/Microbiota-amplicon-bioinformatics/blob/master/Bacteria_16S) and fungal (https://github.com/mhog025/Microbiota-amplicon-bioinformatics/blob/master/Fungi_ITS) bioinformatic pipelines are available online at GitHub. Briefly, primer sequences were removed, bacterial sequence data were trimmed to 250 bp, and the resulting sequences were quality filtered following the parameters outlined in the pipeline. zOTUs were clustered then classified against the SILVA LTP v123 database [29]. Non-target zOTUs, including those that could not be classified to family-level and sequences comprising < 0.1% of the total dataset were removed. Data were normalised to an even depth of 5847 sequences per sample.

Raw fungal sequencing data were processed following the parameters outline in the pipeline on GitHub. Fungal zOTUs were clustered then classified using the UNITE UTAX reference database 10.10.2017 [30]. Non-target zOTUs and those classified as Metazoa or Plantae were removed. Sequences comprising < 0.1% of the total dataset were also removed. Data were normalised to an even depth of 788 sequences per sample.

### Real-time PCR for gene expression

The baseline samples from all four patients and the 7-month post-surgery sample from patient P04 were processed in previous studies [10, 25]. The RNA extracted from these samples were used to extinction and excluded from real-time PCR (RT-qPCR) gene expression analyses. Input RNA volumes for digestion were standardised to 14 µL. Prior to reverse transcription of RNA, any genomic DNA remaining in the RNA extracts was digested using DNase I enzyme following the manufacturer’s instructions (Invitrogen). The presence of any remaining human or bacterial genomic DNA in the RNA extracts after digestion was determined using PCR. The presence of human DNA was assessed by targeting the human ACTB gene with the β-actin F (nucleotide positions 393–413) and β-actin R (nucleotide positions 622–642) primer pair [31, 32]. The presence of bacterial double-stranded DNA was assessed by targeting the V3–V4 regions of the 16S rRNA gene using the S-D-Bact-0341-b-S-17 and S-D-Bact-0785-a-A-21 primers as stated above. A 1 µL input template from each RNA extract was used for PCR. Amplification and thermocycling conditions were as stated above and for both genes. Positive controls comprised either human genomic DNA or *S. aureus* genomic DNA. Negative controls comprised PCR-grade sterile water.

After DNase treatment and assessment of any remaining genomic DNA, the RNA concentration was measured using the NanoDrop™ spectrophotometer. Samples were standardised to 40 ng before RNA reverse transcription reaction to cDNA using SuperScript™ IV VILO Master Mix following the manufacturer’s protocol (ThermoFisher Scientific).

A total of 17 AMR genes, two inflammatory cytokine genes and three housekeeping genes were selected based on previous publications and relevance to CF disease (Additional file 1: Table S3). The expression of each gene was measured in duplicate using a custom TaqMan^®^ array card (ThermoFisher Scientific). RT-qPCR was carried out using the TaqMan^®^Gene Expression Master Mix on the QuantStudio™12K Flex Real-Time PCR System according to the manufacturer’s protocols (ThermoFisher Scientific). The amount of input RNA was standardised so samples and patients could be compared. Raw data were uploaded and analysed using the ‘Relative Quantification’ app on the Connect Data Analysis online ThermoFisher Scientific Cloud [33]. As there were no healthy control samples from patients without CF or patients that were not prescribed antimicrobials, changes to baseline measurements such as these could not be compared. Therefore, normalisation to the endogenous controls genes *18S* (highly expressed), *GAPDH* (moderately expressed), and *HPRT1* (low expression) were used for comparative values within each patient.

### Data analyses

Patient data were collated and reviewed by a clinician. After bioinformatic processing, microbial sequence data were analysed and plotted in R program version 3.6.1 using the R program ‘ggplot2’. For gene expression data, RT-qPCR thresholds were manually set to 0.1 for consistency across targets and array cards. Arithmetic averages of the duplicate C_q_ values were calculated for each sample. The mean ΔC_t_ represents the mean difference between the target C_q_ values and the endogenous control C_q_ values for all the technical replicates for that sample. Values were exported and plotted in R program version 3.6.1.

## Results

Respiratory samples were collected from four patients presenting with CF and CRS at baseline FESS surgery. At least three subsequent samples were collected from each patient in follow-up clinics or revision FESS. Three of the four patients recruited in this study were female, and overall age ranged from 21 to 50 years old at the time of baseline sample collection (Table 1). All patients presented with CRS with nasal polyposis. All four patients were prescribed several long-term medications at the time of recruitment (Additional file 1: Table S1). In the year prior to surgery, patients were prescribed between 3 and 10 antibiotic prescriptions (P01 = 4, P02 = 7, P03 = 10, P04 = 3) (Additional file 1: Table S1). At the time of surgery, P02 was prescribed azithromycin and P04 was prescribed azithromycin and cotrimoxazole (Additional file 1: Table S2).

### Respiratory tract microbiota and AMR determined through cultivation

Culture of sputum and nasal swabs revealed persistent colonisation of respiratory pathogens in all patients. Often, similar species were reported from sputum and nasal cultivation within the same patient at the same time point (Table 2). Culture reports classified bacteria and fungi to species level in many instances. *Staphylococcus aureus*, *Enterobacter cloacae*, and *Pseudomonas aeruginosa* were the most prevalent CF-associated pathogens detected across all samples. Patient P01 had the most diverse sputum and nasal microbiota as determined through cultivation, while the other three patients were mostly characterised by a single species throughout the sampling period.

**Table 2:** Patient sputum and nasal swab cultivation report alongside bacterial 16S rRNA gene sequencing results

Patient	Collection timepoint^b^	Sputum sample	Bacterial 16S rRNA sequencing	Nasal swab	Antimicrobial resistance
P01	Baseline	*Staphylococcus aureus* Other^a^	*Enterobacteriaceae, Enterobacter, Staphylococcus, Corynebacterium*	Other	None assessed
	6	*Staphylococcus aureus, Morganella morganii, Enterobacter cloacae* Other	*Enterobacteriaceae, Enterobacter, Staphylococcus*	*Enterobacter cloacae* Other	Amoxycillin, Amoxycillin + clavulanate, cefuroxime
	13	*Staphylococcus aureus, Chryseobacterium indologenes* Other	*Enterobacteriaceae, Enterobacter, Staphylococcus, Corynebacterium*	*Staphylococcus aureus* Other	Penicillin
	15	*Staphylococcus aureus, mould* Other	*Enterobacteriaceae, Enterobacter, Staphylococcus*	*Enterobacter cloacae, Staphylococcus aureus*	Amoxycillin, cefuroxime, amoxycillin + clavulanate, penicillin
	18	*Staphylococcus aureus, mould* Other	*Enterobacteriaceae, Enterobacter, Staphylococcus*	*Staphylococcus aureus, Enterobacter cloacae*	Penicillin, erythromycin, amoxycillin, cefuroxime, amoxycillin + clavulanate
P02	Baseline	*Pseudomonas aeruginosa* Other	*Staphylococcus, Pseudomonas*	*Pseudomonas aeruginosa* Other	Gentamicin
	2	*Pseudomonas aeruginosa* Other	*Staphylococcus, Pseudomonas*	Other	–
	6	–	*Pseudomonas*	–	–
	11	*Pseudomonas aeruginosa* Other	*Pseudomonas, Corynebacterium*	*Pseudomonas aeruginosa*	Gentamicin
P03	Baseline	*Staphylococcus aureus*	*Staphylococcus*	*Staphylococcus aureus*	Penicillin, flucloxacillin
	3	–	*Staphylococcus, Corynebacterium*	–	–
	6	*Staphylococcus aureus* Other	*Staphylococcus, Enterobacter*	*Staphylococcus aureus*	Penicillin, flucloxacillin
	12	*Staphylococcus aureus* Other	*Staphylococcus, Enterobacteriaceae*	*Staphylococcus aureus*	Penicillin, flucloxacillin
P04	Baseline	Data not collected^c^	*Pseudomonas, Staphylococcus*	*Pseudomonas aeruginosa, Aspergillus fumigatus* Other	Gentamicin, amikacin, ciprofloxacin
	3	–	*Pseudomonas*	*Pseudomonas aeruginosa*	Gentamicin, ciprofloxacin
	6	–	*Pseudomonas*	*Pseudomonas aeruginosa* Other	Ceftazidime, gentamicin
	7	–	*Pseudomonas, Staphylococcus, Corynebacterium*	*Pseudomonas aeruginosa, Fusarium solani complex* Other	Gentamicin, amikacin, ciprofloxacin
	11		*Pseudomonas, Staphylococcus*	*Pseudomonas aeruginosa* Other	Gentamicin

In vitro antimicrobial resistance is noted only for nasal swab isolates. Results from left and right middle meatus swabs for molecular techniques are combined. In vitro antimicrobial resistance profiles from nasal swab bacteria are noted

^a^‘Other’ refers to growth of bacterial colonies that were not considered clinically relevant. This includes mixed oropharyngeal bacteria, mixed Gram-positive and Gram-negative colonies, commensal flora or skin flora. These colonies were not further isolated or identified

^b^Collection timepoint is given as months since baseline collection

^c^Sputum samples were not collected from this patient. Analysis of patient records revealed that prior to lung transplantation in 2012 the dominant organisms recovered from sputum were *Pseudomonas aeruginosa* and *Aspergillus fumigatus*. Sputum collected post lung transplantation, but prior to this study, cultured *Pseudomonas aeruginosa*

Antimicrobial susceptibilities of potential pathogens were conducted in conjunction with cultivation. Along with the highest microbial richness, P01 also had the highest number of observed AMRs in vitro. Both P02 and P04 were characterised by *P. aeruginosa* and reported resistances to gentamicin throughout the sampling period. Patients that were characterised by *S. aureus* were also typically resistant to β-lactam antibiotics such as penicillin, amoxycillin, amoxycillin and clavulanate, and flucloxacillin.

### Microbial profile determined by molecular methods

**Fig. 1:**
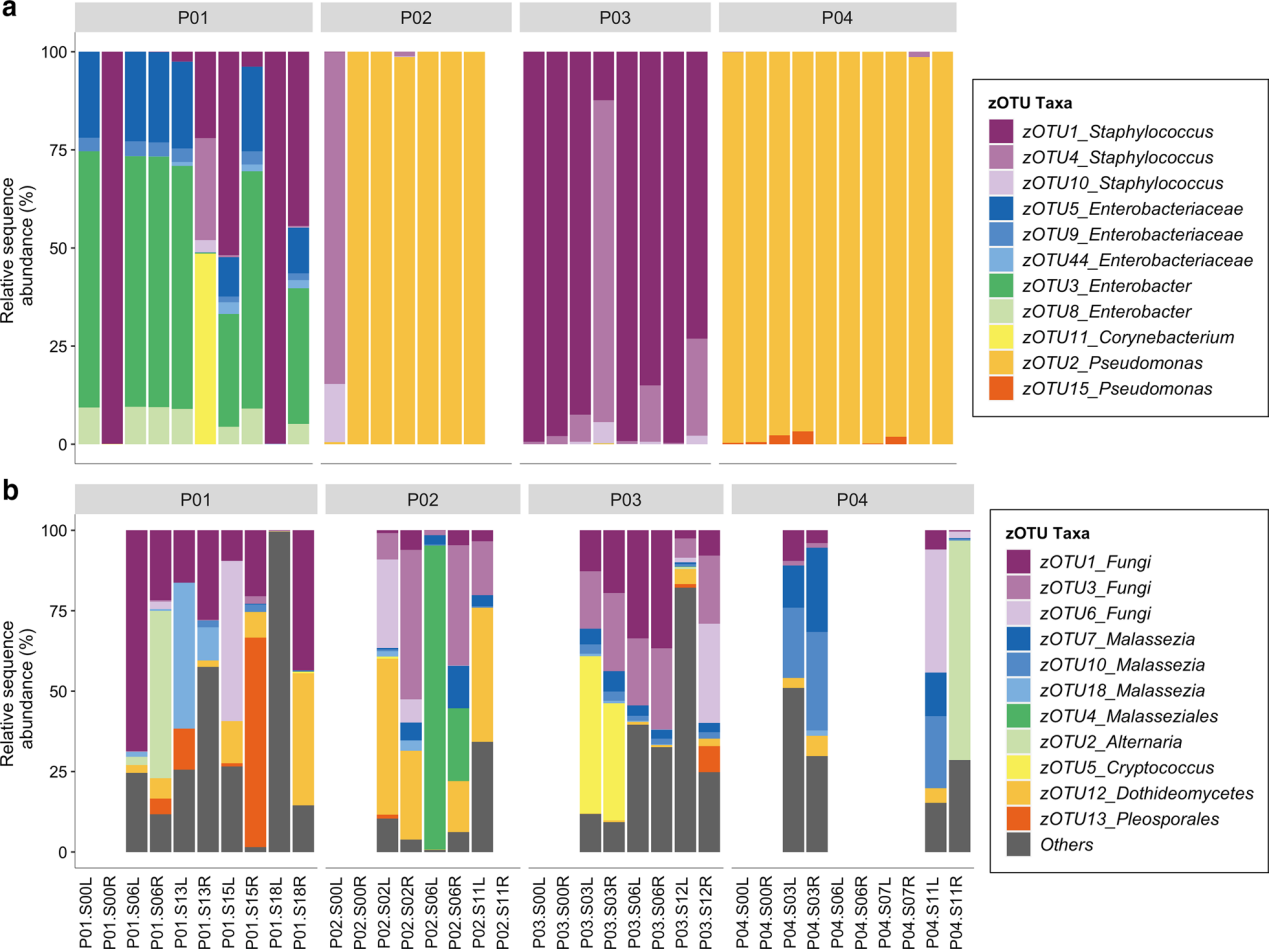
Relative sequence abundance taxa plots of the **a** bacteria and **b** fungi detected from 16S rRNA bacterial gene and fungal ITS2 amplicon sequencing. All detected zero-radius operational taxonomic units (zOTUs) are shown for bacteria, and the top 11 most abundant fungal zOTUs are shown with all remaining combined in “Others”. Missing bars represent samples that did not pass quality filtering steps during bioinformatic analyses. All patient baseline samples and P04 left and right samples 7 months after baseline were excluded from fungal ITS2 sequencing. P = Patient, L = left middle meatus, R = right middle meatus. Months from baseline samples are shown preceding sample side L/R

Rarefaction of bacterial sequencing data resulted in 11 zOTUS detected across all samples. Similar to cultivation results the observed richness overall and within each patient was very low (≤ 9 zOTUs) (Fig. 1). Members of the genus *Pseudomonas* dominated the bacterial community in patients P02 and P04. Sequences assigned to *Enterobacteriaceae* or *Enterobacter* were detected in high relative abundances in P01. Patient P01 also had the highest observed richness of all patients in this study (9 zOTUs observed across an 18-month sampling period). Although the bacterial profiles were fairly consistent through time, patients P01 and P02 saw blooms of *Staphylococcus* zOTUs (Fig. 1a). Patient P03 was characterised by a consistent dominance of *Staphylococcus* spp. throughout the sampling period.

Rarefaction of fungal sequencing data resulted in 64 zOTUs. Members of the genus *Malassezia* and unclassified fungal zOTUs were the most prevalent in the dataset, followed by a long tail of low abundance fungal zOTUs (Fig. 1b). Other fungi including zOTUs belonging to *Aspergillaceae, Aspergillus*, *Alternaria*, *Pleosporales*, *Dothideomycetes*, and *Cryptococcus* were detected in varying relative abundances across patient samples. We observed no patient-specific fungal fingerprint. Fewer fungal amplicon samples passed quality filtering, which suggests these microbes are less abundant than bacteria.

### AMR and inflammatory gene expression

**Fig. 2:**
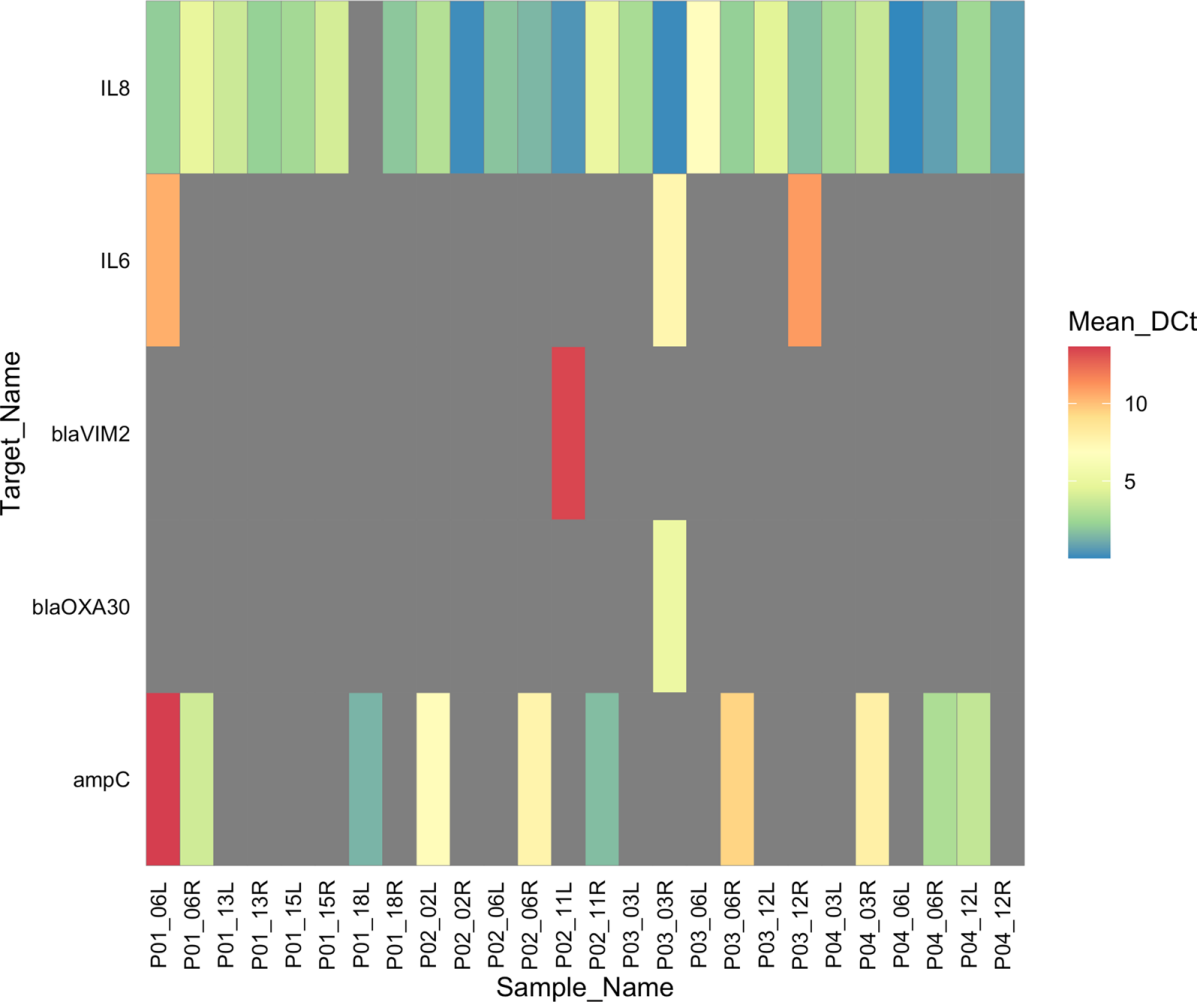
Heatmap depicting the mean delta C_t_ (ΔC_t_) values of antimicrobial resistance and host inflammatory gene expressions from patient sinus swab samples in this study. Elevated expressions of genes, and therefore lower ΔC_t_ values are noted in blue colours, and lower expressions of genes are noted in red. Mean ΔC_t_ values were obtained by calculating the differences between endogenous control genes (18S, GAPDH, HPRT1) and genes of interest. Only those genes which were detected in at least one sample are shown. Samples with no gene expression are shaded in grey. All patient baseline samples and P04 left and right samples 7 months after baseline were excluded from gene expression analyses. Mean_DCt = mean ΔC_t,_ P = Patient, L = left middle meatus, R = right middle meatus. Months from baseline samples are shown preceding sample side L/R

We observed very low or no expression for the selected AMR genes in this study, suggesting that these genes may not be constitutively expressed or the input concentration of RNA was not high enough for detection. For example, when expression of any AMR genes were detected the raw C_t_ values ranged from 33.1–39.4, and maximum C_t_ was 40. Of the 17 genes measured, *ampC*, *blaVIM2*, and *blaOXA30* were detected in at least one sample (Fig. 2). Notably, *ampC* was the only AMR gene expressed in all four patients in at least one sample. Interestingly, variable expressions were observed through time and between sides of the same patient.

The expression of host inflammatory (IL8, IL6) genes were more prevalent and highly expressed compared with the AMR genes. However, similar to AMR gene expression results, we observed variable expressions of these genes in samples from different sides. IL8 expression was detected in nearly all samples, whereas IL6 expression was inconsistent.

## Discussion

Several longitudinal studies have been published that describe changes in the CF lower airway microbiome over time. Fewer studies have evaluated the longitudinal stability of the upper airway microbiome; however, it would appear that the predominant taxa colonising the airways of adult CF patients tend to exhibit remarkable resilience [21, 34–36]. Although some changes may occur during acute exacerbations or treatment, global shifts from these predominant taxa seldom occur in adult CF patients. The results of our study support this observation.

### Long-term microbial profiles: cultivation versus molecular detection

Overall, there was substantial congruence between the nasal swab culture and sinus molecular results taken at the same timepoint within patients. Bacterial species grown in culture from nasal swabs were nearly always detected by bacterial sequencing. However, some key differences were noted. Members from the genus *Corynebacterium* were detected typically in low abundances in the molecular data, however, none were reported in cultivation. This could be because *Corynebacterium* spp. are typically commensal bacteria, less likely to impact CF disease and are either not reported or targeted for by specific diagnostic culture methods. Nasal swab culture reports mirrored the most abundant organisms detected by bacterial sequencing. Furthermore, sputum culture reported similar results as those from nasal swab cultivation and bacterial sequencing at the same timepoint within patients. A few differences were observed. For example, *Morganella morganii* and *Chryseobacterium indologenes* were detected in the sputum of patient P01 but not in nasal swab cultivation. Neither of these organisms were detected in the quality filtered dataset, however, inspection of the pre-filtered zOTU table revealed members from the *Morganella* family were detected in P01 at the same timepoint. We recommend that futures studies utilising sequencing techniques carefully examine filtering parameters for low abundant taxa, as these low abundant taxa may be clinically relevant.

Fungal growth was rarely reported in these samples, so the possible comparisons to molecular results were few. It is likely that routine microbiological culture techniques do not target fungi, especially if fungal infection is not suspected. Mould was reported in two of the sputum samples from P01 but further taxonomic classification was not given. *Aspergillus fumigatus* was detected in the baseline nasal swabs from P04. Two zOTUs were assigned to family *Aspergillaceae* and two to genus *Aspergillus*. These four zOTUs were detected in several samples from P01, P03, and P04. Members from the *Fusarium solani* complex were reported in culture, however, these were not detected in the molecular dataset.

Fungi are typically detected in the upper and lower airways during health, chronic airway disease, and acute infections [9, 25]. In health, fungi are likely a transient part of the microbiome and inter-kingdom interactions remain understudied. Fungi have been detected in sinus culture reports from CF patients, but infrequently [37]. A diverse array of fungi have been detected in the CF airways in general, including, *Alternaria* spp., *Cladosporium* spp., *Malassezia* spp., *Aspergillus* spp., *Candida* spp., and many others [38, 39]. Similar to potential bacterial pathogens, the presence of fungal isolates does not necessarily infer invasive disease. While bacterial infections often characterise CF lung decline, clinical manifestations associated with fungal detections vary ranging from asymptomatic colonisation to sensitisation, hypersensitivity and invasive disease [39].

### Antimicrobial prescription, AMR and host gene expression

The selection of AMR bacteria is a significant concern for CF patients. Very few studies have investigated the expression of AMR genes in sinus samples from CF patients, and little is known of the stability of these genes in the upper airways. In our study, we measured the expressions of 17 AMR genes and resistance profiles of cultivated pathogens in vitro. Although many of the bacterial isolates were multidrug resistant when tested by antimicrobial diffusion discs in vitro, we did not see high levels of AMR gene expression in situ. One reason could be that the patients in this study were sampled while clinically stable. Another explanation is that the thick mucus in the sinuses of CF patients may reduce the exposure of bacteria to antibiotics and reduce the pressure for microbes to constitutively express AMR genes [40]. Lastly, we hypothesise that elevated expression of other AMR genes may be detected with higher input concentrations of RNA. The comparatively low proportion of bacterial RNA versus higher levels of human gene expression could mask lower levels of bacterial RNA. However, higher input concentrations on our array card in this study would have meant that the detection of highly expressed host genes would have exceeded the detectable range.

The RT-qPCR results showed that the *ampC* gene was expressed in low levels in all patients, but at varying timepoints and inconsistently between left and right sinuses. Activation of and mutations within this gene confers resistance to most penicillins, cephalothin, cefazolin, cefoxitin, β-lactamase inhibitor-β-lactam combinations, and broad spectrum cephalosporins [41]. Members of *Enterobacteriaceae* and *P. aeruginosa* are known to carry mutations in this gene. Our bacterial sequencing results and culture reports noted the presence of these organisms, and their suspected AMR activity was confirmed in the in vitro analyses.

The multifaceted approach in this study allows for investigations between cultivated isolate in vitro AMR, in situ expressions of AMR genes, and antimicrobial prescriptions to patients at the time of each sample collection. For example, in P01 at the 6-month sample collection, increased expression of *ampC* was detected in the sinuses. The sequencing results suggested bacteria in the *Enterobacteriaceae* family and *Enterobacter* spp. dominated the sinuses and this observation was supported by cultivation of *Enterobacter cloacae* in both the sinuses and sputum. In vitro AMR reports suggests the *E. cloacae* isolate was resistant to amoxycillin, amoxycillin + clavulanate, and cefuroxime. The expression of *ampC* is consistent with *Enterobacteriaceae* bacteria, and the amoxycillin + clavulanate prescribed at the time would induce expression of *ampC* [41]. This patient was previously treated with cotrimoxazole, doxycycline and roxithromycin in the year prior to sample collection which are typically effective against *Enterobacter* spp. At the 13-month timepoint, a*mpC* expression is not detected in the sinuses. The cultivation reports suggested the *Enterobacter* colonisation cleared, and instead *S. aureus* was cultured and detected in the sequencing. *S. aureus* is not known to have *ampC*, and no expression was detected. Following on, *ampC* expression typically coincided with detection of *Enterobacter* spp. *AmpC* is also known to occur in *Pseudomonas* spp., and in this study we saw expression of *ampC* occurring with detection of *Pseudomonas* in culture and sequencing data in P02 and P04. Specifically, P02 and P04 are prescribed ongoing daily and weekly doses of the macrolide antibiotic azithromycin for treatment of *P. aeruginosa* respiratory exacerbations.

Numerous pro-inflammatory cytokines (IL-1β, IL6, IL8, and TNFα) are elevated in the lower airways of CF patients compared to healthy controls [15]. However, some evidence suggests that the immune response in the nasal mucosa of CF patients is not as severe as that observed in the lower airways [42]. This may partially explain why we observed inconsistent expression of IL6 in the sinus swabs, in contrast to previous studies on the lower airways. Cell culture experiments with nasal epithelial cells from CF patients showed significantly increased levels of IL8 under basal conditions, and even higher expressions when challenged with *P. aeruginosa* [43]. Our results support previous observations of consistently elevated expression of IL8. Future studies should compare cytokine expression levels in both the upper and lower airways of the same patients through time.

### Study limitations

This pilot study is limited by the small number of patients and therefore the results may not be broadly applicable to other CF patients. However, extensive collection of patient demographic and clinical data, and the range of culture and molecular techniques applied across several timepoints are a real strength. We observed that microbial and inflammatory gene expression profiles were relatively stable throughout the sampling timepoints, findings which are consistent with previous studies. This stability likely reflects the clinical stability of patients during each sampling timepoint. However, the very different sampling timepoints in our study may impact data analyses and interpretation. Future longitudinal studies should endeavour to sample at regular timepoints as more nuanced changes in the microbiome can be detected. We also recommend that future studies include younger patients to capture the dynamic nature of the upper airway microbiota prior to surgical intervention. Sample collection before, during and after CF and CRS acute exacerbations requiring antibiotic treatment will offer valuable insights into the changes in AMR gene expression.

Our results support the previous observation that molecular methods are more sensitive than culture for detecting microbial communities. However, DNA-based sequencing techniques do not differentiate between actively replicating and dead microbes. Additionally, the strain-level variability in CF patients is high and sequencing such short fragments of the bacterial 16S rRNA gene cannot provide conclusive species- or strain-level taxonomic assignments. We recommend that future studies consider using strain-typing of isolates in order to capture subtle but potentially important variations in bacterial strains. The combination of culture dependent and independent techniques applied in this study can serve as a framework for future longitudinal airway microbiome studies. Collection of additional patient information, such as consumption of non-prescribed medication, including probiotics, could help with interpretation of results in larger studies.

### Support for a unified airway microbiome

Endoscopic sinus surgery may alter the relative abundances of the host’s microbial profile, but surgery does not eradicate the microbiota altogether [44]. In a previous study, similar *P. aeruginosa* isolates that colonised the lungs and sinuses of CF patients prior to lung transplantation were later detected in the new grafts [5]. In our study, many of the bacterial profiles were consistent before and after surgery. These results may be a reflection of the advanced stage of disease, but they also lend support to the unified airway theory. Although it is hypothesised that microbes from the upper airways transfer to the lower airways, the direction of translocation has yet to be proven [45].

## Conclusions

Providing comprehensive treatment strategies that target both the upper and lower airways is important for reducing CF morbidity. The study presented here provides a framework for subsequent larger studies that aim to evaluate the role of the upper and lower microbiome during CF. The results of this study advance our understanding of the stability of the upper airway microbiota of CF patients. These data suggest that the upper airway microbial communities are stable through long periods of time in patients post endoscopic sinus surgery. We observed sustained increased expressions of IL8 and lower, frequent expressions of the AMR gene *ampC*. Comprehensive longitudinal studies are required to expand upon these observations and provide a solid foundation for assessing the impacts of new gene targeted treatments on the unified airway during CF disease progression.

## Supplementary Information

**Additional file 1:** **Table S1.** Patient long-term medication and antibiotic prescription history. **Table S2. **Patient antibiotic prescription at each sampling timepoint. **Table S3.** A list of the AMR genes, tight junction and inflammatory host genes measured in this study using the custom TaqMan^®^ Gene Expression Assay array card.

## Data Availability

Due to the sensitive nature of data, the raw sequencing data associated with this study are available by request from the corresponding author.

## Abbreviations

AMR: Antimicrobial resistance
CF: Cystic fibrosis
CRS: Chronic rhinosinusitis
FESS: Functional endoscopic sinus surgery
ITS2: Internal transcribed spacer region 2
PCR: Polymerase chain reaction
16S rRNA: 16S ribosomal ribonucleic acid
RT-qPCR: Quantitative reverse transcription polymerase chain reaction
zOTU: Zero-radius operational taxonomic unit
