# Supplementary material for: Longitudinal analysis of sinus microbiota post endoscopic surgery in patients with cystic fibrosis and chronic rhinosinusitis: a pilot study
Source: Respir Res. 2021 Apr 13;22:106. doi: 10.1186/s12931-021-01697-w (PMC8045235; doi:10.1186/s12931-021-01697-w)
Supplement: Supplementary file 1 — Additional file 1: Table S1. Patient long-term medication and antibiotic prescription history. Table S2. Patient antibiotic prescription at each sampling timepoint. Table S3. A list of the AMR genes, tight junction and inflammatory host genes measured in this study using the custom TaqMan® Gene Expression Assay array card. [file 12931_2021_1697_MOESM1_ESM.docx]

**Additional files**

**Table S1**. Patient long-term medication and antibiotic prescription history.

| **Patient** | **Long-term medications at the time of surgery** | **Number of antibiotic prescriptions year prior to baseline collection** | **Antibiotic prescription at baseline** | **Type** |
| --- | --- | --- | --- | --- |
| P01 | 1. Creon 10,000, 15 daily 2. Flixonase 1 each nostril daily  3. Sinus rinse 4. Hypertonic saline 7% 4 ml nebulised bd 5. Vitamin D monthly | 4 | No | No |
| P02 | 1. Creon Forte 13 od 2. VitABDECK 2 tablets daily (Special Authority CHEM/108601714/Life) 3. Pulmozyme 2.5 mg nebulised daily 4. Fortisip 12 scoops daily  5. Bricanyl Turbuhaler 2 puffs bd 6. Azithromycin 250 mg daily on weekdays 7. Nebulised hypertonic saline 7% 4 mL bd 8. Alanase nasal spray 9. Symbicort 200/6 two puffs bd  10. Calcium carbonate 1.25 g daily  11. Cholecalciferol 1.25 mg/month | 7 | Yes | Azithromycin |
| P03 | 1. Salbutamol inhaler 100 µg 2 puffs prn 2. Beclomethasone intranasal spray 2 puffs bd (Alanase 100 µg) 3. Cholecalciferol tablets 50,000 units monthly  4. Omeprazole 40 mg bd. (30 minutes prior to breakfast and dinner) 5. Seretide 125/25 inhaler 2 puffs bd 6. Loratadine 10 mg nocte 7. Flixotide 125 µg 2 puff bd  8. Multivitamins 2 daily 9. Laxsol 2 tablets prn 10. Movicol prn 11. Nasal rinses prn 12. Tramadol 50 mg od./bd  13. Zoladex injection 3 monthly 14. Provera 1 per day 15. Atorvastatin 40 mg per day 16. Ranitidine 300 mg nocte 17. Metformin 500 mg 2 bd | 10 | No | No |
| P04 | 1. Tacrolimus 2.5 mg bd (dose reduced) 2. Mycophenolate 750 mg bd  3. Prednisone 5 mg daily  4. Nasal saline washes with baking soda daily  5. Cotrimoxazole 400/80 mg Tuesday and Friday 6. Omeprazole 20 mg bd 7. Creon 10000 20 tablets daily prn 8. Azithromycin 250 mg three times per week 9. Melatonin 3 mg nocte prn 10. Vitamin D six tablets stat then one tablet monthly | 3 | Yes | Azithromycin; Cotrimoxazole |

**Table S2.** Patient antibiotic prescription at each sampling timepoint.

| **Patient** | **Sample Collection** | **Antibiotic prescription** | **Type** |
| --- | --- | --- | --- |
| P01 | Baseline | No | - |
|  | 6m | Yes | Amoxicillin + clavulanic acid |
|  | 13m | Yes | Doxycycline |
|  | 15m | No | - |
|  | 18m | Yes | Azithromycin |
| P02 | Baseline | Yes | Azithromycin |
|  | 2m | Yes | Azithromycin |
|  | 6m | Yes | Azithromycin |
|  | 11m | Yes | Azithromycin |
| P03 | Baseline | No | - |
|  | 3m | No | - |
|  | 6m | Yes | Cotrimoxazole, Amoxicillin + clavulanic acid |
|  | 12m | No | - |
| P04 | Baseline | Yes | Cotrimoxazole, Azithromycin |
|  | 3m | No | - |
|  | 6m | Yes | Cotrimoxazole, Azithromycin |
|  | 7m | Yes | Cotrimoxazole, Azithromycin |
|  | 11m | Yes | Cotrimoxazole, Azithromycin |

**Table S3.** A list of the AMR genes, tight junction and inflammatory host genes measured in this study using the custom TaqMan® Gene Expression Assay array card.

| **Gene** | **Target name** |
| --- | --- |
| *CXCL8* | IL8 |
| *IL6* | IL6 |
| *blaNDM-1* | Metallo-β-lactamase |
| *blaOXA-30* | β-lactamase (Class D) |
| *ampC; FOX* | AmpC β-lactamase (Class C) |
| *blaGES-3* | Carbapenemase (Class A) |
| *blaKPC-3* | Carbapenemase (Class A) |
| *blaIMP; blaIMP-4* | Carbapenemase (Class B) |
| *blaOXA-162; blaOxa-48* | Carbapenemase (Class D) |
| *blaPER-1* | Extended Spectrum β-lactamase (Class A) |
| *blaVEB-1* | Extended Spectrum β-lactamase |
| *blaCTX-M-3* | Extended Spectrum β-lactamase |
| *blaCTX-M-35* | Extended Spectrum β-lactamase |
| *blaCTX-M-63* | Extended Spectrum β-lactamase |
| *blaCTX-M-64; blatoho-3* | Extended Spectrum β-lactamase |
| *erm;ermB* | Macrolide Resistance |
| *qnrA2* | Quinolone Resistance |
| *qnr* | Quinolone Resistance |
| *blaVIM-2* | Verona integron-encoded metallo-β-lactamase |
| *18S* | Endogenous control |
| *GAPDH* | Endogenous control |
| *HPRT1* | Endogenous control |
